# Commentary: Survey of Areas Underserved by Plastic Surgery in Japan

**DOI:** 10.1055/s-0043-1777241

**Published:** 2024-02-22

**Authors:** Makoto Sato

**Affiliations:** 1 Department of Plastic Surgery, Hyogo Prefectural Nishinomiya Hospital, Hyogo, Japan

Survey of Areas Underserved by Plastic Surgery in Japan

In Japan, there is a large regional disparity in plastic surgery availability. In order for plastic surgery to be widely available for all citizens, it is essential for at least one plastic surgery facility to be located in each secondary medical zone. Using the Japan Society of Plastic and Reconstructive Surgery homepage and some databases, we extracted data on secondary medical zones that do not have a plastic surgery facility. The national and regional coverage rates were calculated. The coverage rate for each group divided by the degree of population concentration was also calculated. We found that 147 of 344 secondary medical zones did not have a plastic surgery facility, and the area coverage rate was found to be 57.27% nationwide. The coverage rate in terms of population was 87.07% (correlation coefficient of area and population coverage = 0.983). The area coverage rates in Hokkaido-Tohoku, Kanto, Chubu, Kansai, Chugoku-Shikoku, and Kyushu-Okinawa districts were 47.46, 72.15, 76.47, 62.79, 52.08, and 32.81%, respectively. The corresponding population coverage rates were 79.92, 91.62, 94.27, 90.59, 80.68, and 69.54%, respectively. The area coverage rates in metropolitan areas, provincial cities, and rural areas were 98.08, 75.90, and 15.87%, respectively. In contrast, the area coverage rate of dermatology was 62.79% and that of orthopaedics was 97.09%. Unfortunately, it is estimated that more than 40% of secondary medical zones are underserved by plastic surgery, and 13% of the population is not able to fully benefit from this specialty in Japan.



